# Big Data Analytics for Scanning Transmission Electron Microscopy Ptychography

**DOI:** 10.1038/srep26348

**Published:** 2016-05-23

**Authors:** S. Jesse, M. Chi, A. Belianinov, C. Beekman, S. V. Kalinin, A. Y. Borisevich, A. R. Lupini

**Affiliations:** 1 The Institute for Functional Imaging of Materials, Oak Ridge National Laboratory, Oak Ridge, TN 37831, USA; 2 The Center for Nanophase Materials Sciences, Oak Ridge National Laboratory, Oak Ridge, TN 37831, USA; 3 Florida State University Department of Physics Tallahassee, FL 32306, USA; 4 National High Magnetic Field Laboratory, 1800 E Paul Dirac Dr, Tallahassee, FL, 32310, USA; 5 Materials Sciences and Technology Division, Oak Ridge National Laboratory, Oak Ridge, TN 37831, USA.

## Abstract

Electron microscopy is undergoing a transition; from the model of producing only a few micrographs, through the current state where many images and spectra can be digitally recorded, to a new mode where very large volumes of data (movies, ptychographic and multi-dimensional series) can be rapidly obtained. Here, we discuss the application of so-called “big-data” methods to high dimensional microscopy data, using unsupervised multivariate statistical techniques, in order to explore salient image features in a specific example of BiFeO_3_ domains. Remarkably, k-means clustering reveals domain differentiation despite the fact that the algorithm is purely statistical in nature and does not require any prior information regarding the material, any coexisting phases, or any differentiating structures. While this is a somewhat trivial case, this example signifies the extraction of useful physical and structural information without any prior bias regarding the sample or the instrumental modality. Further interpretation of these types of results may still require human intervention. However, the open nature of this algorithm and its wide availability, enable broad collaborations and exploratory work necessary to enable efficient data analysis in electron microscopy.

Scanning transmission electron microscopy (STEM) and associated spectroscopies have emerged as powerful tools for the visualization of structure and functionality of materials at atomic resolution^1^,^2^. The widespread implementation of aberration correction, and the associated increase in spatial resolution has allowed sub-50 pm resolution and, consequently, determination of atomic positions with sub-10 pm precision^3^,^4^,^5^,^6^. These capabilities enable direct visualization of chemical and mechanical strains^7^, order parameter fields including ferroelectric polarization^8^,^9^,^10^,^11^, and octahedral tilts^12^,^13^,^14^,^15^,^16^. Additional opportunities are enabled by more sophisticated analysis of the images, with examples including extracting atomic column shapes, which have been shown to contain information with regards to otherwise inaccessible parameters, like the tilt system in the beam direction^16^,^17^,^18^. In a related development, the increase in convergence angle (necessary for improved transverse resolution) also enables focal series imaging, yielding three-dimensional images of materials structure. Similar information has been obtained from the reconstruction of tilt series^17^ and the combination of these two techniques in a “tilt-focal series”^18^ appears extremely promising. However, in all these cases, the image contrast originates from one or two (e.g. bright field and dark field) information channels sampled over 2D (imaging) or 3D (series) spatial grids. Therefore, the structural information is inferred by a mathematical analysis under implicit assumptions about the image formation mechanisms. For example, maxima in high-angle annular dark field (HAADF) image intensities are usually identified as atomic columns, and hence analysis of contrast provides enhanced information regarding atomic positions.

A typical STEM imaging detector integrates the response over a range of scattering angles (e.g. high-angle scattering). However, the fundamental physics of the image formation mechanism in STEM offers a significantly richer source of information in the form of a local diffraction image also refered to as a Ronchigram^19^. The term Ronchigram is rather loosely defined, as it sometimes refers to the STEM shadow map without an aperture. All of the diffraction patterns discussed here have an angle-defining aperture, we therefore use the term Convergent Beam Electron Diffraction (CBED) pattern throughout the rest of the manuscript. CBED patterns offer far more information because instead of integrating over a range of scattering angles to arrive at a single representative data point, the whole scattering distribution is recorded. Previous theoretical and experimental works suggest that full acquisition of the CBED patterns at each spatial location in a scan can enable super resolution, phase-contrast imaging, as well as imaging of internal fields, and 3D sample reconstruction^20^,^21^,^22^,^23^.

Traditionally, problems in attempting to access the complete dataset were threefold: first, limitations imposed by the detector acquisition speed, second the data storage demands, and third, processing, synthesis and visualization of the of the data to extract useful information. In the last several years, data acquisition and storage have evolved to a point where it is now possible to capture and save high-resolution multi-dimensional data sets rapidly. However, the underlying complexity and dearth of mathematical tools used to visualize and analyze these data are compounded by the nebulous information content of the data itself. The basic assumptions about the image formation process might precondition the expectation of what information is available. Alternatively, to put it bluntly, prior expectations might both limit the potential information that can be extracted^24^ and create false positives for expected results^25^,^26^,^27^. Here, we describe a comprehensive framework for processing and mining of large (multi-GB) data sets, to distil the most salient aspects of the data while separating out the statistically significant variations from noise to hopefullyextract useful information about the material being examined,. Here, we will discuss the extraction of the physically-relevant parameters based on the statistically relevant similarities of CBED patterns. Furthermore, we deliberate on a roadmap for data streaming and storage for ptychographic imaging of complex materials.

## Results

### Experimental Details

**Figure 1:**
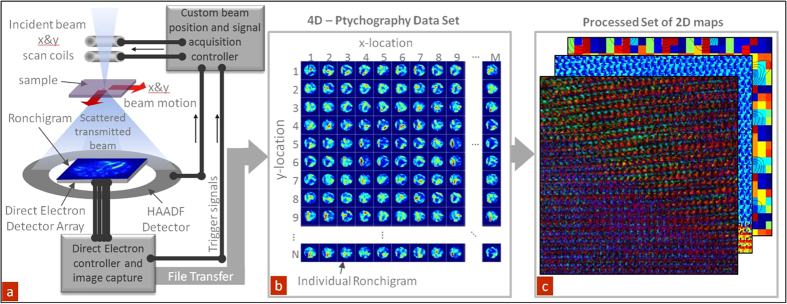
Schematic of the data acquisition system. (**a**) The control system synchronizes beam positioning and image capture from a direct electron detector of the CBED pattern and recording of the HAADF signal. (**b**) The resulting 4D data set (where a 2D CBED pattern is captured at each spatial location across a M by N 2D array of points) is analyzed to construct (**c**) 2D maps showing variations in material properties across the samples.

To enable rapid acquisition of the CBED data, we utilized a DE-12 camera (Direct Electron, LP, San Diego, CA), equipped with a 4096 × 3072 pixels Direct Detection Device (DDD^®^) sensor installed on an aberration corrected FEI Titan operating at 300 kV. The camera and the microscope were integrated through a custom FPGA control system to synchronize frame capture and beam positioning to acquire 4D scanning-scattering data sets. The schematic of the data acquisition system is illustrated in Fig. 1a. In this specific example, we raster scanned the electron beam over 192 × 192 physical positions on the sample, collecting 384 × 384 pixel CBED patterns at each beam location. We have utilized a capture rate of ~300 frames per second to give a total acquisition time approximately 1 minute for the entire 4D dataset. We note that this acquisition speed is comparable to STEM spectrum imaging and hence allows (in principle) a transition to “full data” acquisition imaging in STEM using existing instrumental infrastructure once the associated data streaming pipelines and data analytic tools are established, similar to the approach recently demonstrated for scanning probe microscopy^28^,^29^,^30^. For the analysis presented here, the CBED pattern data was binned down to 96 × 96 pixel images (from the full 384 × 384) to enable calculation on a desktop computer. However, high-performance computational environments will enable this methodology for much larger data sets.

From a physical perspective, the data set is stored as a 4D array in the form *S(x*, *y*, *u*, *v)*, where *S* is the measured signal intensity, *x*, *y* are the coordinates of the electron beam in the image plane (probe position), and *u*, *v* are the coordinates in the k-space of the system in the camera detector plane (detector pixel or angle). For a combined ptychographic focal-series dataset, the dimensionality increases to 5D, taking on the form *S(x*, *y*, *z*, *u*, *v)*, where *z* is the focal plane of the beam. The multidimensional nature of the data necessitates the development of systematic ways to easily explore the associated structure in real and reciprocal-spaces, with real time analytics, analysis and visualization.

We chose a highly strained polymorph bismuth ferrite (BFO) thin film as a model system for this study. The thin film was grown by PLD on a LaAlO_3_ substrate, forming coexisting T’ and S’ phases. Detailed growth condition and phase information were described previously^31^. As these two neighboring phases have identical chemical composition, but different crystal symmetry^32^, their phase boundaries exhibit interesting interfacial phenomena, such as local elastic and electric susceptibilities, which are confined at a small length scale but may lead to unique physical properties^33^,^34^. A precise two-dimensional map of the structure at atomic scale across the phase boundary is prerequisite to unravel the complex correlations between interfacial structure and physical properties in this system.

We note, as a first step of the analysis it is helpful to emulate the response of a bright field or a low angle annular dark field detector by calculating the mean intensity of the specific regions of the 4D CBED pattern dataset (*u*, *v*) at all scan locations (*x*, *y*). Such representations allow rapid, qualitative assessment of the data, including resolution and drift, as well as an overview of material structure (e.g. the presence of topological and structural defects, dissimilar phases, etc.) (see supplementary Figure 1). Once these basic relationships are established one can take a more in-depth, statistical look at the whole dataset.

For periodic and nearly-periodic systems, an initial insight into the structure of the data can be obtained by using fast a Fourier transform (FFT) on the spatial coordinates, i.e. the transformation of *S(x*, *y*, *u*, *v)* to *S(ρ*_*x*_, *ρ*_*x*_, *u*, *v)*, where *ρ* is used to indicate spatial frequency.

**Figure 2:**
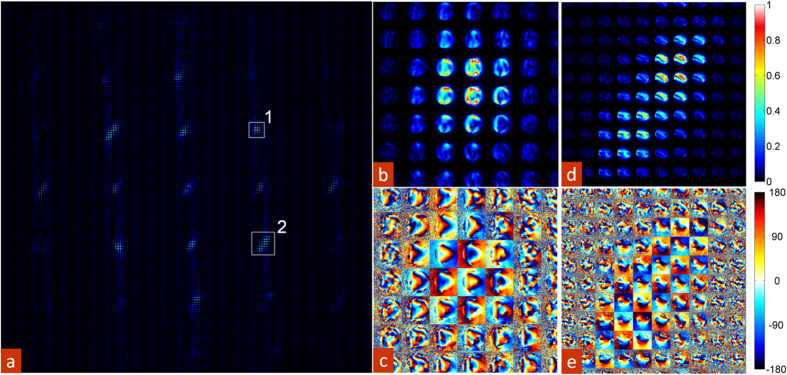
Fourier analysis of the 4D scanning-scattering data set. (**a**) Fourier transforming across the spatial dimensions allows one to view periodicity in the structure of the sample as well as view the CBED patterns associated with a specific spatial periodicity. (**b**) Amplitude and (**c**) phase of the transformed CBED patterns located at peak 1 in (**a**). (**d**) Amplitude and (**e**) phase of the transformed CBED patterns located at peak 2 in (**a**).

Figure 2 illustrates the FFT of the multidimensional data set for the BFO sample. Here, the coordinate system in the image corresponds to the reciprocal lattice vectors of the main lattice, whereas each (compound) pixel represents the characteristic CBED pattern at a particular spatial frequency. Note that if the CBED pattern information is averaged to a single pixel, this information is reduced to the classical FFT of an image, with clearly visible maxima corresponding to the inverse lattice vectors of the material. However, detailed examination of the data illustrates the rich internal structure of the data set, as visualized in Fig. 2b–e. The CBED pattern amplitude information of peaks labeled 1 & 2 in Fig. 2a, is shown in detail in panels b & d, with the phase shown in Fig. 2c,e. The phase images (Fig. 2c,e) contain information on the details of the aberrations and illumination coherence^35^. Furthermore, FFT on a 4D dataset shows clear peak splitting of the <210> peak, highlighted by a square and label “2” in Fig. 2a with a zoomed view in Fig. 2d, similar to a 2D FFT of BFO. The 2D equivalent has been used to capture material crystal orientation, asses the quality of the grown material, as well as ferroelectric domains in relevant materials^36^. However, the 4D representation enables access to individual CBED patterns, which can be selected, averaged and inversely Fourier transformed to spatially map a given orientation’s contribution to the overall image. In addition, the phase portion of the signal, shown in Fig. 2e, can serve as a quick quantitative assessment of the lattice strain difference between different orientations, and potentially provide information on polarization. This fusion of classical analysis with modern data capabilities enables entirely new ways of interpreting results and pushing the limits of instrumentation to at least qualitatively asses, hitherto inaccessible properties.

### Multivariate Statistical Methods

To gain further insight into the structure and information content of the ptychographic data set, we performed Principal Component Analysis (PCA) following the framework developed earlier for the reflection of high energy electron diffraction (RHEED) and STM data sets^37^,^38^,^39^. Here, the original 4D data set is reshaped into a 2D data set of size *P* × *Q*, where the total number of spatial locations (*N*_*x*_ × *N*_*y*_) = *P*, and the total number pixels in the CBED pattern (*N*_*u*_ × *N*_*v*_) = *Q*. The resultant 2D data set, ***D***, is decomposed using conventional principal component analysis^40^,^41^,^42^,^43^,^44^,^45^,^46^.

In PCA, defined by Equation (1), a spectroscopic data set of *P* populated by spectra containing *Q* points is represented as a weighted superposition of the eigenvectors ***V***, in Equation 1
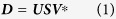
where the cross-product, ***US***_*i*,*j*_, are the expansion coefficients at each pixel. The eigenvectors ***V*** and the corresponding eigenvalues *S* are calculated with a covariance matrix, ***C*** = ***DD***^T^, where ***D*** is the matrix of all experimental data points ***D***_***i***,***j***_, i.e. the rows of ***D*** correspond to individual scan positions (*i* = 1,…,*P*), and columns correspond to a point in a CBED pattern, ( *j* = 1,…,*Q*). The eigenvectors ***V*** are orthogonal and are ordered so that the eigenvalues are placed in descending order, *λ*_1_ > *λ*_2_ > …. Hence, the first eigenvector, ***V***_***1***,***j***_, contains the most information (where information is defined as variance) within the spectral-image dataset; the second contains the most “informative” (varying) response after the subtraction of the first one, and so on. In this manner, the first *q* loading maps, ***U***_***i***,***1:q***_, contain the majority of information within the 3D dataset, while the remaining *Q*-*q* sets are dominated by highly uncorrelated information which is likely to be noise.

**Figure 3:**
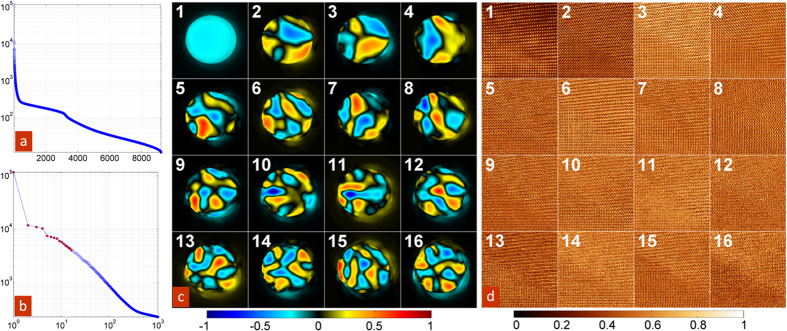
PCA analysis. (**a**) Full log-linear scree plot of information content for all (96 × 96) 9216 principal components. (**b**) Log-log scree plot of the first 1000 principal components with the first 16 corresponding to images in (**b**,**c**) highlighted. (**c**) The first 16 PCA eigenvectors. (**d**) The first 16 PCA loading maps.

The resulting 2D matrices are converted back to the real space and detector coordinates, yielding data sets of the form ***U***_*i*_(*x*, *y*) and ***V***_*i*_(*u*, *v*). The measure of variance associated with each loading map ***U***_*i*_(*x*, *y*) is taken from values ***S***_***i***_,_***i***_ and are used to generate scree plots (Fig. 3a,b). The ***U***_*i*_(*x*, *y*) are the PCA loading maps, representing spatial variation of the CBED patterns between dissimilar probe locations in terms of linear combinations of eigenvectors ***V***_*i*_(*u*, *v*). Note, while PCA components are defined in a purely statistical sense and generally do not have well defined physical meaning (unless the structure of decomposition is identical to the physics of the system, as can be the case for e.g. Bayesian unmixing^47^,^48^,^49^), they do provide insight into the variability of the response and the information content of the ptychographic data set. In particular, unlike compound real-space and FFT images, (because each pixel contains a 2D CBED pattern), PCA allows representation of spatially dependent information in the form of a set of 2D images; which allows identification of large scale structural features, and individual morphological elements that are statistically significant within a given data set.

The amount of information in a ptychographic data set can be estimated based on the shape of the scree plot, shown in Fig. 3a,b. In this case, the inflection point is located at approximately 300 principal components, suggesting that ~300 components out of 9216 contain relevant information. The behavior of the PCA components for the BFO is illustrated in Fig. 3d. The first PCA component, which by definition is equivalent to the average signal, since we have utilized raw, non-whitened data, effectively represents a bright field image of the material. Interestingly, some higher order components show a differentiation between the two regions of dissimilar phases (visible gradations of the intensity between the domains); with some cases (component 11) exhibiting contrast at the interface region. We note that while physical interpretation of individual eigenvectors beyond symmetries can be challenging, as is the case with the original CBED patterns, this approach allows for high-veracity visualization and structural examination of material structure as a purely statistical dissection based on the signal variance. Although PCA does not transform the data into components with direct physical meaning (owing primarily to the underlying eigenvector orthonormality constraint of the method), this statistical approach excels at compressing and de-noising large data sets very rapidly. For these reasons, PCA can serve as an effective initial step to clean and reduce the data to a more manageable size while maintaining the information rich content in preparation for more computationally intensive analysis steps downstream. More importantly, we are reducing the data based on a statistical evaluation of quality and content, rather than the traditional averaging route, void of any discrimination.

Analysis of the unfolded 4D to 2D data sets can be further extended to explore similarities and patterns in materials structure via clustering analysis. For example, the k-means algorithm can be used to divide *M* points in *N* dimensions into *K* clusters in such a way as to minimize the variance within each cluster, Equation (2)
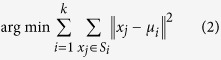
where ***μ***_***i***_ is the mean of points in *S*_*i*_^50^,^51^. Here, we have used a Matlab k-means algorithm that minimizes the sum, over all clusters, of the within-cluster sums of point-to-cluster-centroid distances. The measure of distance (the minimization parameter) is in square-Euclidian space with each centroid being the component-wise median of the points in a given cluster.

**Figure 4:**
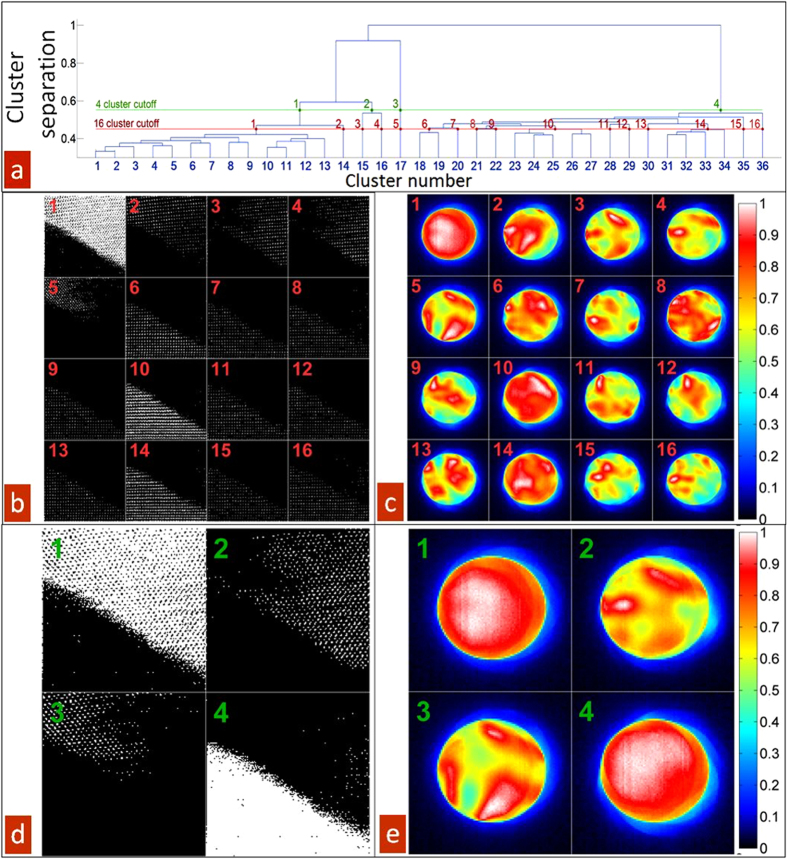
K-mean cluster analysis results showing hierarchical organization and spatial distribution of clustered CBED patterns. (**a**) Dendrogram showing the hierarchical clustering from 36 clusters to a single one. Vertical length of branches indicates relative separation distance between neighboring clusters. The red line shows the cut-off for generating 16 clusters as shown in (**b**,**c**), and the green lines shows the cut-off for generating 4 clusters as shown in (**d**,**e**). Binary spatial maps indicating the locations of clusters associated with a particular cluster for (**b**) 16 and (**c**) 4 total clusters. Mean CBED patterns associated with each cluster for (**c**) 16 and (**d**) 4 total clusters.

The k-means approach requires the k-value (the number of clusters) entered a-priori, and it is therefore a challenge to initially determine an appropriate number of clusters that best represents the data. To this end, we used a relatively large value for k = 36 (which is higher than necessary, and will be reduced later), and applied a k-means clustering method on the first 256 elements of the weighting vectors for all spatial points (***U***_1..P,1..256_) see Supplemental Material Figure S1. Figure 4a shows the top-down agglomerative hierarchical cluster tree (dendrogram) assembled from the clustering branches by determining the closest two clusters, combining them into one cluster, and continuing until only one cluster remained. The relative distance between cluster centroids is represented by the height of the vertical drop at which two clusters are joined in the dendrogram. Therefore, this plot offers a convenient representation of separations and grouping within the data. The structure of the dendrogram clearly illustrates a progressive division into two main branches and one sub-branch. The main branches then generate a continuum of cluster states. The spatial localization of resultant clusters is illustrated in Fig. 4, as shown for the 4 (green) and 16 (red) level division. Clusters 1–36, are shown in Supplementary Materias Figure S2. Note that the initial decomposition (clusters 1, 2, 3 belonging to first branch and cluster 4 belonging to second branch) clearly separates the S’ and T’ phase of BFO, where the orientation between the c-axis of the two phase should be within 5 degrees.

**Figure 5:**
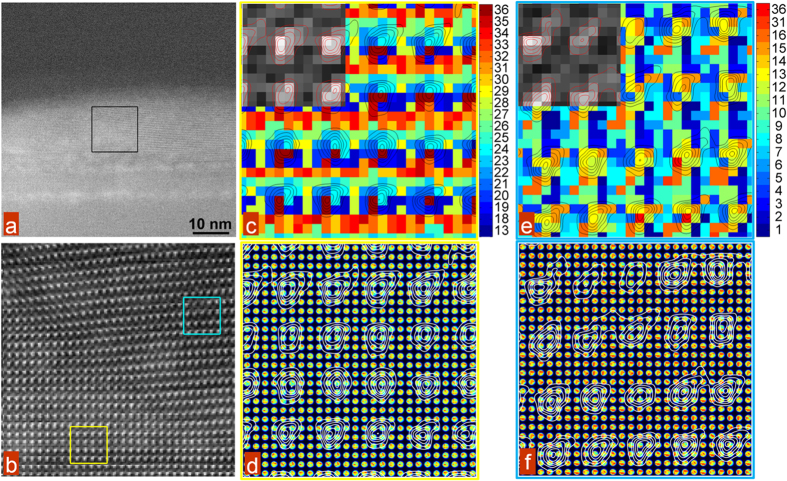
Atomic scale, and CBED analysis of fine atomic structure. (**a**) An overview of the BFO film area where atomic resolution measurements were taken - illustrated by a black square. (**b**) The zoomed in HAADF image of the area shown by a black square in (**a**). (**c**) A subset of the image shown in (**b**), highlighted by a yellow square, illustrating a 36 cluster k-means result performed on the CBED patterns. Atomic positions are overlaid as contour maps, the original HAADF pixel values are shown in the upper left. (**d**) The same area as (**c**) shown with the original CBED patterns at each pixel. Atomic positions are shown as contour maps. (**e**) A subset of the image shown in (**b**), highlighted by a blue square, illustrating a 36 cluster k-means result performed on the CBED patterns. Atomic positions are overlaid as contour maps, the original HAADF pixel values are shown in the upper left. (**f**) The same area as (**e**) shown with the original CBED patterns at each pixel. Atomic positions are shown as contour maps.

The subsequent clustering visualizes the variability of the ptychographic data set on the atomic level, effectively sorting the scattering information at atomic resolution. Note, that the resultant clusters are localized within phases (as can be expected based on the hierarchical character of clustering process). The high-resolution images are shown in Fig. 5. Note the periodicity of the cluster distribution, indicating that specific clusters are associated with specific probe positions in relation to the atomic columns. In other words, the mean of each cluster represents the typical CBED pattern from specific points within the unit cell. Additionally, regular tiling of cluster arrangements commensurate with unit cell spacing provides a means to reveal, in a systematic way, the effects of local fields on electron scattering behavior.

## Discussion

We note that the analysis described above presents a starting framework for the systematic analysis of the ptychographic data sets, enabling exploration of underlying materials structure, identification of the relevant materials behaviors, and compression for storage and analysis. The latter can include data analytics using models that incorporate the physics of measurement process (e.g. linear unmixing with superimposed physical constraints), numerical detectors optimized for specific physics, and direct comparison with libraries of simulated data for solution of inverse problems.

We also note that the transition to ptychographic imaging should potentially enable super resolution imaging^19^. Similarly, since the potential information content of a single 300 × 300 pixel CBED pattern is much higher than for a single integrated value, there will be important consequences for the signal to noise ratios. Obviously while this view is oversimplified and ignores noise sources (such as electron flux and 1/*f* noises in the system) it illustrates the potential for ptychographic imaging and may suggest possible directions for theoretical analyses and additional technique development. HAADF imaging is often the preferred mode in a STEM, because it is typically simple to interpret without much sample information. On the other hand, BF imaging may be more sensitive to subtle changes in the electron phase, and for thin, light materials will have a far larger flux of electrons per solid angle than high-angle scattering under equivalent conditions. Therefore, an approach capable of combining the sensitivity of the BF imaging mode with the minimal prior knowledge requirements of the HAADF imaging could have far reaching consequences.

Finally, we briefly analyse the physical data infrastructure requirements for ptychographic imaging. Today, we already have the capability to capture much larger 4D datasets for multiple thousands of probe positions and CBED patterns resolved at 4 k by 4 k pixels with the newest high-pixel-count electron detectors. Practically, however, using 32 bit integers, a thousand points in every dimension ((1 k × 1 k probe positions) × (1 k × 1 k CBED pattern resolution)) results in a 4 TB dataset. To sustain such gargantuan data output streams, ideally the microscope data would be livestreamed directly from the instrument to a large database associated with sufficent computational power. Initial insight presented in this work serves to develop efficient compression algorithms at the data generation point to ameliorate these requirements.

We can also consider how much information is available given a finite number of electrons in the probe. To account for the spatial beam coordinates and scattering angle of each electron, we would need approximately 16 bytes. For a very-high resolution STEM, a typical probe current might be around 32 pA, translating to roughly 200 electrons per microsecond and resulting in a data generation rate of 3.2 GB/sec. Obviously, precise values are debatable, since the probe positions may be generated from a systematic function, only the maximal detected intensity might be useful, or electron energy loss spectra might also be recorded. Additionally this data could be a function of frame, or focus, or some physical parameter (time, focal series, tilt series, etc.) adding dimensionality and size, in which case higher transfer rates or more storage capacity would be necessary. These speeds are in-line with the current commercial connection speeds, with dedicated centres routinely having access to optical-fiber connections that operate in the Gb/s regimes. Similarly, although storage space and data access are also nontrivial requirements for such data volume generation, those issues have mostly been addressed with the rise of cloud-based services. A more difficult case is the total memory and processing power available instantaneously, since for some analysis processes the bottleneck is in the availability of random access memory, rather than raw CPU speed. The processor time to calculate full a PCA decomposition using a current workstation (Intel Xenon E5-1650V3, 32GB DDR3 RAM) for the CBED BFO dataset is approximately 30 minutes, however the RAM requirement to hit that benchmark is 625GB. Alternatively, the k-means clustering process is CPU limited taking almost entire 24 hours to complete. Clearly, in order to process much larger datasets a high performance computing (HPC) environment with scalable analysis code that is capable of transfer, storage and fast analysis of multidimensional data sets is vital.

## Additional Information

**How to cite this article**: Jesse, S. *et al*. Big Data Analytics for Scanning Transmission Electron Microscopy Ptychography. *Sci. Rep.* **6**, 26348; doi: 10.1038/srep26348 (2016).

## Supplementary Material

Supplementary Information

## References

[b1] Pennycook S. J. & Nellist P. D. Scanning Transmission Electron Microscopy: Imaging and Analysis (Springer, New York, 2011).

[b2] Pennycook S. J. . In Advances in Imaging and Electron Physics, Vol. 153 Advances In Imaging and Electron Physics (ed. Hawkes P. W. ) 327 (Elsevier Academic Press Inc, 2008).

[b3] Kisielowski C. . Detection of single atoms and buried defects in three dimensions by aberration-corrected electron microscope with 0.5-Å information limit. Microsc. Microanal. 14, 469–477 (2008). 18793491 10.1017/S1431927608080902

[b4] Yankovich A. B. . Picometre-precision analysis of scanning transmission electron microscopy images of platinum nanocatalysts. Nat. Commun. 5, doi: 4155, 10.1038/ncomms5155 (2014). 24916914

[b5] Kim Y. M. . Probing oxygen vacancy concentration and homogeneity in solid-oxide fuel-cell cathode materials on the subunit-cell level. Nat. Mater. 11, 888–894, doi: 10.1038/nmat3393 (2012). 22902896

[b6] Sawada H. . STEM imaging of 47-pm-separated atomic columns by a spherical aberration-corrected electron microscope with a 300-kV cold field emission gun. *J. Electron Microsc.*, dfp030 (2009). 10.1093/jmicro/dfp030 19546144

[b7] Kim Y. M. . Direct observation of ferroelectric field effect and vacancy-controlled screening at the BiFeO3/LaxSr1-xMnO3 interface. Nat. Mater. 13, 1019–1025, doi: 10.1038/nmat4058 (2014). 25129618

[b8] Chang H. J. . Atomically Resolved Mapping of Polarization and Electric Fields Across Ferroelectric/Oxide Interfaces by Z-contrast Imaging. Adv. Mater. 23, 2474–2479, doi: 10.1002/adma.201004641 (2011). 21538586

[b9] Nelson C. T. . Spontaneous Vortex Nanodomain Arrays at Ferroelectric Heterointerfaces. Nano Lett. 11, 828–834, doi: 10.1021/nl1041808 (2011). 21247184

[b10] Jia C. L. . Unit-cell scale mapping of ferroelectricity and tetragonality in epitaxial ultrathin ferroelectric films. Nat. Mater. 6, 64–69, doi: 10.1038/nmat1808 (2007). 17173031

[b11] Jia C. L., Urban K. W., Alexe M., Hesse D. & Vrejoiu I. Direct Observation of Continuous Electric Dipole Rotation in Flux-Closure Domains in Ferroelectric Pb(Zr,Ti)O(3). Science 331, 1420–1423, doi: 10.1126/science.1200605 (2011). 21415348

[b12] Jia C. L. . Oxygen octahedron reconstruction in the SrTiO(3)/LaAlO(3) heterointerfaces investigated using aberration-corrected ultrahigh-resolution transmission electron microscopy. Phys. Rev. B 79, doi: 081405 10.1103/PhysRevB.79.081405 (2009).

[b13] Kim Y. M. . Interplay of Octahedral Tilts and Polar Order in BiFeO3 Films. Adv. Mater. 25, 2497–2504, doi: 10.1002/adma.201204584 (2013). 23505214

[b14] Borisevich A. Y. . Suppression of Octahedral Tilts and Associated Changes in Electronic Properties at Epitaxial Oxide Heterostructure Interfaces. Phys. Rev. Lett. 105, doi: 087204, 10.1103/PhysRevLett.105.087204 (2010). 20868130

[b15] He J., Borisevich A., Kalinin S. V., Pennycook S. J. & Pantelides S. T. Control of Octahedral Tilts and Magnetic Properties of Perovskite Oxide Heterostructures by Substrate Symmetry. Phys. Rev. Lett. 105, doi: 227203, 10.1103/PhysRevLett.105.227203 (2010). 21231419

[b16] Borisevich A. . Mapping Octahedral Tilts and Polarization Across a Domain Wall in BiFeO(3) from Z-Contrast Scanning Transmission Electron Microscopy Image Atomic Column Shape Analysis. ACS Nano 4, 6071–6079, doi: 10.1021/nn1011539 (2010). 20919690

[b17] Midgley P. & Weyland M. 3D electron microscopy in the physical sciences: the development of Z-contrast and EFTEM tomography. Ultramicroscopy 96, 413–431 (2003). 12871805 10.1016/S0304-3991(03)00105-0

[b18] Dahmen T. . Combined Scanning Transmission Electron Microscopy Tilt-and Focal Series. Microsc. Microanal. 20, 548–560 (2014). 24548618 10.1017/S1431927614000075

[b19] Rodenburg J. Ptychography and related diffractive imaging methods. Adv. Imaging Electron Phys. 150, 87–184 (2008).

[b20] Godden T., Suman R., Humphry M., Rodenburg J. & Maiden A. Ptychographic microscope for three-dimensional imaging. Opt. Express 22, 12513 (2014). 24921369 10.1364/OE.22.012513

[b21] Humphry M., Kraus B., Hurst A., Maiden A. & Rodenburg J. Ptychographic electron microscopy using high-angle dark-field scattering for sub-nanometre resolution imaging. Nat. Commun. 3, 730 (2012). 22395621 10.1038/ncomms1733 PMC3316878

[b22] Müller K. . Atomic electric fields revealed by a quantum mechanical approach to electron picodiffraction. Nat. Commun. 5 (2014). 10.1038/ncomms6653 PMC4275586 25501385

[b23] Pennycook T. J. . Efficient phase contrast imaging in STEM using a pixelated detector. Part 1: Experimental demonstration at atomic resolution. Ultramicroscopy 151, 160–167 (2015). 25458189 10.1016/j.ultramic.2014.09.013

[b24] MacCoun R. & Perlmutter S. Blind analysis: Hide results to seek the truth. Nature 526, 187–189 (2015). 26450040 10.1038/526187a

[b25] Kalinin S. V., Sumpter B. G. & Archibald R. K. Big-deep-smart data in imaging for guiding materials design. Nat. Mater. 14, 973–980 (2015). 26395941 10.1038/nmat4395

[b26] Chisholm M. F. . Comment on” Single Crystals of Single-Walled Carbon Nanotubes Formed by Self-Assembly”. Science 300, 1236–1236 (2003). 12764177 10.1126/science.1080395

[b27] Lupini A. R. . Letter to the Editor: Limitations to the Measurement of Oxygen Concentrations by HRTEM Imposed by Surface Roughness. Microsc. Microanal. 11, 111–113 (2005). 15817138 10.1017/S1431927605210309

[b28] Belianinov A., Kalinin S. V. & Jesse S. Complete information acquisition in dynamic force microscopy. Nat. Commun. 6, doi: 655010.1038/ncomms7550 (2015). 10.1038/ncomms7550 25766370

[b29] Collins L. . Multifrequency spectrum analysis using fully digital G Mode-Kelvin probe force microscopy. Nanotechnology 27, 105706 (2016). 26866505 10.1088/0957-4484/27/10/105706

[b30] Somnath S., Belianinov A., Kalinin S. V. & Jesse S. Full information acquisition in piezoresponse force microscopy. Appl. Phys. Lett. 107, 263102 (2015).

[b31] Beekman C. . Phase transitions, phase coexistence, and piezoelectric switching behavior in highly strained BiFeO3 films. Adv. Mater. 25, 5561–5567 (2013). 23847158 10.1002/adma.201302066

[b32] Christen H. M., Nam J. H., Kim H. S., Hatt A. J. & Spaldin N. A. Stress-induced R− M A− M C− T symmetry changes in BiFeO 3 films. Phys. Rev. B 83, 144107 (2011).

[b33] Seidel J. . Prominent electrochromism through vacancy-order melting in a complex oxide. Nat. Commun. 3, 799 (2012). 22531184 10.1038/ncomms1799

[b34] Zeches R. . A strain-driven morphotropic phase boundary in BiFeO3. Science 326, 977–980 (2009). 19965507 10.1126/science.1177046

[b35] Lupini A., Wang P., Nellist P., Kirkland A. & Pennycook S. Aberration measurement using the Ronchigram contrast transfer function. Ultramicroscopy 110, 891–898 (2010). 20434843 10.1016/j.ultramic.2010.04.006

[b36] Burch M. J., Li J., Harris D. T., Maria J.-P. & Dickey E. C. Mechanisms for microstructure enhancement in flux-assisted growth of barium titanate on sapphire. J. Mater. Res. 29, 843–848, doi: 10.1557/jmr.2014.59 (2014).

[b37] Vasudevan R. K., Tselev A., Baddorf A. P. & Kalinin S. V. Big-Data Reflection High Energy Electron Diffraction Analysis for Understanding Epitaxial Film Growth Processes. ACS Nano 8, 10899–10908, doi: 10.1021/nn504730n (2014). 25268549

[b38] Vasudevan R. K. . Big data in reciprocal space: Sliding fast Fourier transforms for determining periodicity. Appl. Phys. Lett. 106, 091601 (2015).

[b39] Belianinov A. . Research update: spatially resolved mapping of electronic structure on atomic level by multivariate statistical analysis. APL Mater. 2, 120701 (2014).

[b40] Bosman M., Watanabe M., Alexander D. T. L. & Keast V. J. Mapping chemical and bonding information using multivariate analysis of electron energy-loss spectrum images. Ultramicroscopy 106, 1024–1032, doi: 10.1016/j.ultramic.2006.04.016 (2006). 16876322

[b41] Bonnet N. In Advances in Imaging and Electron Physics, Vol. 114 Advances In Imaging and Electron Physics (ed. Hawkes P. W. ) 1–77 (Elsevier Academic Press Inc, 2000).

[b42] Bonnet N. Multivariate statistical methods for the analysis of microscope image series: applications in materials science. J. Microsc. (Oxford, UK) 190, 2–18, doi: 10.1046/j.1365-2818.1998.3250876.x (1998).

[b43] Jesse S. & Kalinin S. V. Principal component and spatial correlation analysis of spectroscopic-imaging data in scanning probe microscopy. Nanotechnology 20, 085714, doi: 10.1088/0957-4484/20/8/085714 (2009). 19417475

[b44] Belianinov A. . Big data and deep data in scanning and electron microscopies: deriving functionality from multidimensional data sets. ASCI 1, 1–25 (2015). 10.1186/s40679-015-0006-6 PMC4977326 27547705

[b45] Iberi V. . Graphene engineering by neon ion beams. Nanotechnology 27, 125302 (2016). 26890062 10.1088/0957-4484/27/12/125302

[b46] Belianinov A. . Identification of phases, symmetries and defects through local crystallography. Nat. Commun. 6 (2015). 10.1038/ncomms8801 PMC4518243 26190623

[b47] Strelcov E. . Deep Data Analysis of Conductive Phenomena on Complex Oxide Interfaces: Physics from Data Mining. ACS Nano 8, 6449–6457, doi: 10.1021/nn502029b (2014). 24869675

[b48] Strelcov E., Belianinov A., Hsieh Y.-H., Chu Y.-H. & Kalinin S. V. Constraining data mining with physical models: voltage-and oxygen pressure-dependent transport in multiferroic nanostructures. *Nano Lett*. 15(10), 6650–6657, doi: 10.1021/acs.nanolett.5b02472 (2015). 26312554

[b49] Tselev A. . Mapping internal structure of coal by confocal micro-Raman spectroscopy and scanning microwave microscopy. Fuel 126, 32–37 (2014).

[b50] Hartigan J. A. & Wong M. A. Algorithm AS 136: A K-Means Clustering Algorithm. J. Appl. Stats. 28, 100–108, doi: 10.2307/2346830 (1979).

[b51] MacQueen J. B. In Proc. of the fifth Berkeley Symposium on Mathematical Statistics and Probability Vol. 1 (eds Le CamL M. & Neyman J. ) 281–297 (University of California Press, 1967).

