# Supplementary material for: Big Data Analytics for Scanning Transmission Electron Microscopy Ptychography
Source: Sci Rep. 2016 May 23;6:26348. doi: 10.1038/srep26348 (PMC4876439; doi:10.1038/srep26348)
Supplement: Supplementary Information [file srep26348-s1.pdf]

Supplemental Material

Big Data Analytics for Scanning Transmission Electron Microscopy Ptychography

S. Jesse,[1,2] M. Chi,[1,2] A. Belianinov,[1,2] Christianne Beekman,[3] S.V. Kalinin,[1,2] A. Borisevich,[1,4] and A. Lupini[1,4]

[1] The Institute for Functional Imaging of Materials Oak Ridge National Laboratory, Oak Ridge, TN 37831

[2] The Center for Nanophase Materials Sciences Oak Ridge National Laboratory, Oak Ridge, TN 37831

[3] Florida State University, 1800 E Paul Dirac Dr., Tallahassee FL 32310

[4]Materials Sciences and Technology Division, Oak Ridge National Laboratory, Oak Ridge, TN 37831

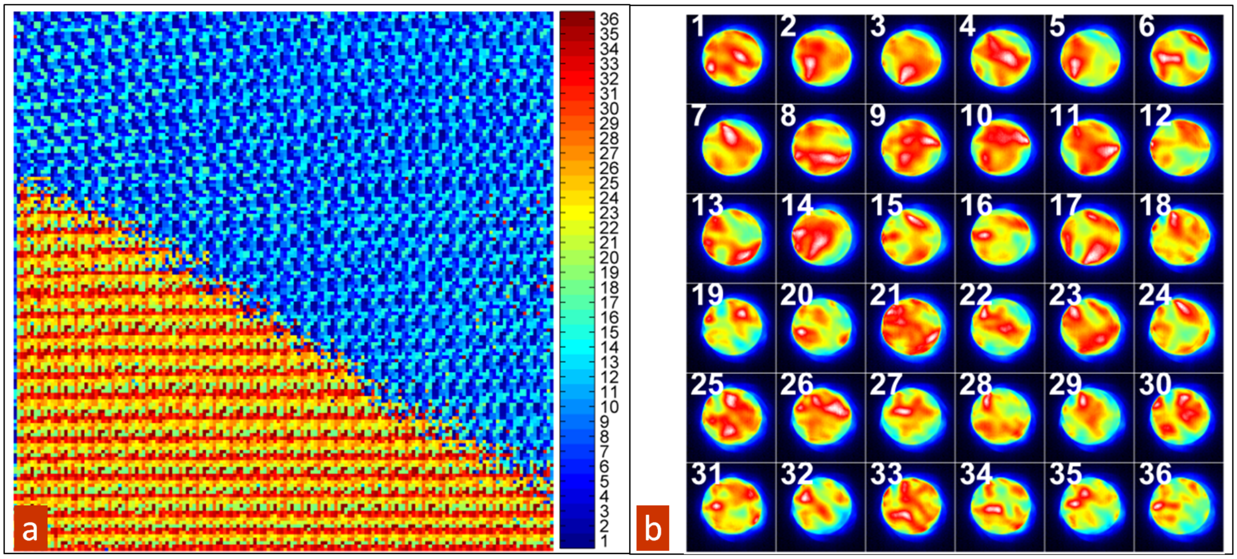

Supplemental Material Figure 1: K-means clustering results. (a) BFO data visualized with 36 clusters. (b) Individual Ronchigrams describing each of the clusters visualized

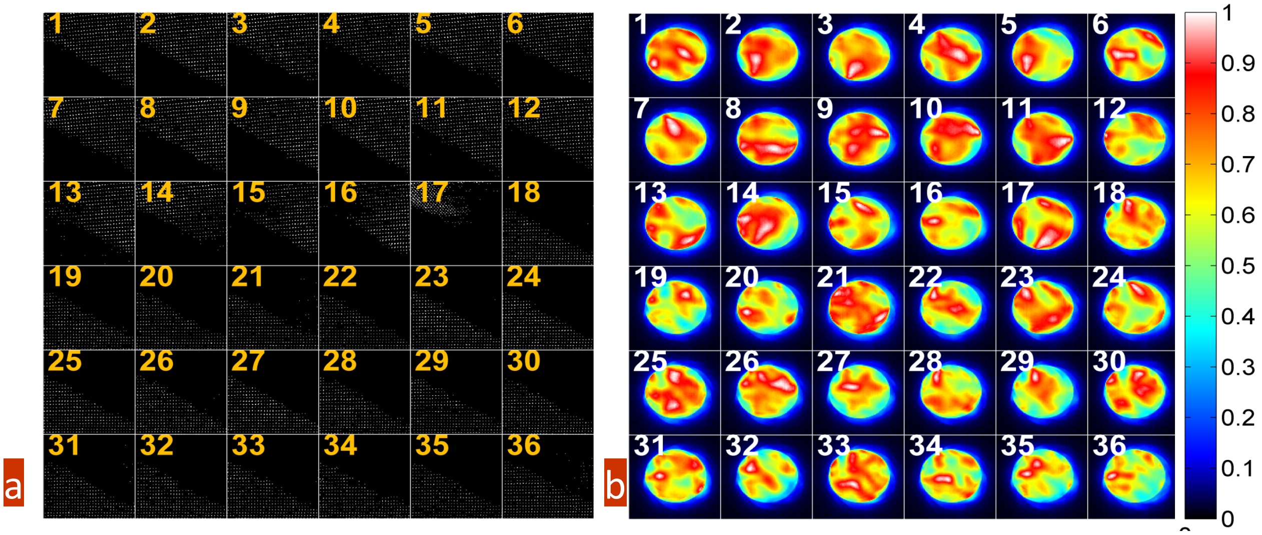

Supplemental Material Figure 2: K-means clustering results. (a) BFO data separated into individual 36 clusters. (b) Individual Ronchigrams describing each of the clusters visualized
